# Effects of Different Concentrations of AmB on the Unsaturated Phospholipid–Cholesterol Membrane Using the Langmuir Monolayer and Liposome Models

**DOI:** 10.3390/molecules29235659

**Published:** 2024-11-29

**Authors:** Juan Wang, Jia Wang, Mingyue Zheng, Da Li

**Affiliations:** 1 Xi’an Key Laboratory of Advanced Photo-Electronics Materials and Energy Conversion Device, School of Electronic Information, Xijing University, Xi’an 710123, China; 2 Shaanxi Engineering Research Center of Controllable Neutron Source, School of Electronic Information, Xijing University, Xi’an 710123, China

**Keywords:** Amphotericin B, membrane of erythrocyte, Langmuir monolayer, liposome, BAM

## Abstract

Amphotericin B (AmB) causes toxicity to the erythrocyte membrane, leading to hemolysis, which limits the clinically effective dose for AmB intravenous therapy in invasive fungal infections. The molecular mechanism by which AmB adheres to the membrane of erythrocytes is the key factor in causing AmB to be toxic to the membrane of erythrocytes, but it is not yet fully understood; the mechanism by which AmB adheres to the liquid microdomains with higher fluidity formed by cholesterol and unsaturated phospholipids remains especially unclear. This study examined the adsorption of AmB at different concentrations, 5, 45, 85, and 125 μg/mL, on unsaturated phospholipid membranes containing 50 mol% cholesterol. The thermodynamic properties and structure of DOPC monolayers and DOPC/cholesterol mixed monolayers at different concentrations of AmB have been investigated using the Langmuir monolayer model and the BAM method. The impact of varying concentrations of AmB on the hydrophilic and hydrophobic domains of the DOPC bilayers and the DOPC/cholesterol mixed bilayers have also been discussed using large unilamellar vesicle liposomes and fluorescence techniques. It is shown that for AmB concentrations greater than 5 μg/mL, with an increase in AmB’s concentration, the reorganization time for the DOPC/cholesterol monolayer increases, and the elastic modulus of the DOPC/cholesterol mixed monolayer decreases. In particular, when AmB’s concentration is higher than 85 μg/mL, the liquid-condensed phase domains on the DOPC/cholesterol monolayer reduce significantly and the liquid-expanded phase domain enlarges from the BAM images. When the AmB concentration reaches 5 μg/mL, the disorder of the hydrophobic and hydrophilic domains of the DOPC/cholesterol bilayer increases as the AmB concentration increases. The way in which AmB interacts with the DOPC/cholesterol mixed membrane is related to the concentration of AmB. The higher the concentration of AmB, the more likely it is to remove cholesterol from the unsaturated phospholipid membrane. The results are helpful to understand the mechanism of AmB’s toxicity to the erythrocyte’s membrane, which has a guiding value for seeking ways to reduce the AmB’s toxicity.

## 1. Introduction

In recent years, there has been an increase in the prevalence and incidence of invasive fungal infections, with an elevated mortality rate associated with the disease [1]. Amphotericin B (AmB) has emerged as the gold standard for managing invasive fungal infections in clinical settings due to its broad-spectrum antifungal action and low incidence of clinical drug resistance [2,3]. It is a macrolide antibiotic [4], comprising a rigid lactone ring formed by carbon atoms and an aminoglycoside component [5]. The lipophilic heptacene chromophore and hydrophilic polyol fragment constitute the amphipathic nature of the molecule within the lactone ring (the molecular structure is shown in Section 3.1), while the polar groups of AmB are represented by the aminoglycoside component and the carboxylic acid at position C16 [6]. The molecular structure, with multiple chains, can engage in non-specific interactions with the lipid components of cell membranes, such as ergosterol or cholesterol [7,8]. The non-specific binding of AmB renders it highly toxic to the membrane of mammalian cells, including the erythrocyte membrane.

The primary role of erythrocytes is to facilitate the transfer of oxygen from the pulmonary system to diverse bodily tissues. Hemoglobin, responsible for oxygen transport, and the membrane of erythrocytes collectively exert a predominant influence on the regulation of gas exchange. AmB can bind to cholesterol on the cell membrane, leading to the formation of membrane pores [9], and this is no exception for the erythrocyte membrane. These pores enhance the permeability of the erythrocyte’s membrane, leading to the leakage of hemoglobin and other essential substances, thereby exacerbating the damage to the erythrocyte’s structure and impairment of its function [10]. After the membrane of the erythrocyte is damaged, some alterations in cell morphology may occur, including an increase in spherocytes and irregularly shaped erythrocytes. The membrane elasticity of erythrocytes is significantly impaired due to the morphological changes, as demonstrated by Sokolova et al.’s dynamic study of hypo-osmotic hemolysis of native and AmB-modified erythrocytes [11]. Damage to the membrane elasticity of erythrocytes will negatively affect the physiological function of erythrocytes [12]. The AmB molecules can modify the hemolytic permeability of the erythrocyte’s membrane [13,14], which causes the hematotoxicity and restricts the clinical dosage of this drug. Some scholars have been directing its attention towards the investigation of AmB liposome agents [15] and AmB derivatives with low toxicity [16,17], but the effect of reducing toxicity is not obvious. The fundamental issue stems from the lack of clarity regarding the molecular mechanism of AmB’s adsorption on the membrane of erythrocytes, which also is a key factor contributing to the toxicity on the erythrocyte’s membrane induced by the AmB drug.

At present, it is generally believed that there may be two models of the membrane toxicity mechanism of amphotericin B—“pore” assumption and “sponge” assumption. In the “pore” assumption [18,19], the AmB molecules bind to sterols to form single and double water pore channels on the cell membrane; a small number of AmB molecules may aggregate and arrange in a V-shape, forming non-aqueous pores. In the “sponge” assumption, AmB molecules form an extramembrane spongy aggregate and attach to the surface of the cell membrane, which can extract sterols from the membrane, thus damaging the structure and function of the cell membrane [9,20]. In both mechanisms, sterols are a key target for the toxicity of AmB to the cell membrane. Cholesterol is a class of sterols widely found in mammalian cell membranes. It is a type of lipid involved in lipid rafts [21], and it also acts as a regulator for signaling molecules and functional proteins. The proportion of cholesterol in different types of cell membranes varies depending on their different function. It is highly enriched in the plasma membrane (PM), accounting for about 30 mol% of all lipids on the membrane [22,23], and up to 50 mol% of total membrane lipids in the membrane of tumor cells and erythrocytes [24,25,26]. In addition, in the absence of cholesterol, AmB can interact with phospholipids on the cell membrane through hydrophobic interactions to form supramolecular complexes, thus forming non-aqueous pores [27,28,29]. Meanwhile, the interaction between AmB and cholesterol is influenced by the phospholipid components present in the membrane [30,31,32]. The pore formation kinetics of AmB are directly related to the thickness and fluidity of phospholipid membrane, and are also affected by the saturation degree of the tail chain of phospholipids [33]. Therefore, in order to understand the toxicity of AmB to erythrocyte membranes, it is necessary to identify the interaction between AmB and the cholesterol-rich (50%) phospholipid membrane region.

On the cell membrane, cholesterol tends to associate more readily with saturated phospholipids, which cause the formation of functional lipid rafts within the cell membrane [34]. Many scholars have utilized the saturated phospholipid–cholesterol model membrane to investigate the interaction between AmB and biological membranes [35,36,37]. These studies indicate that the fungal amino group of AmB is situated in the hydrophilic region of the 1,2-dipalmitoyl-sn-glycero-3-phosphocholine (DPPC) monolayer, with the longest axis of the AmB molecule perpendicular to the surface of the monolayer. In fact, the phospholipid constituents of the cellular membrane exhibit remarkable complexity, and the manner in which AmB interacts with the phospholipid–cholesterol membrane is influenced by the degree of saturation of phospholipids [38]. Cholesterol can interact with unsaturated phospholipids to form the liquid membrane region [39], exhibiting higher fluidity compared to lipid rafts [40,41]. The changes of the membrane’s fluidity can seriously affect the biological function of natural membranes, including the material transport and cell division [42]. Therefore, identifying the mechanism of interaction between AmB and the liquid membrane composed of cholesterol and unsaturated phospholipids is valuable for understanding the membrane toxicity of AmB.

Due to the complexity of cell membrane components, the membrane components can be effectively controlled by using model membranes, which are conducive to obtaining information about the interaction between AmB and specific components. The lipid bilayer in vesicles and the Langmuir monolayer are usually used as the simplified models of the cell membrane system in vitro. The lipid bilayer of vesicles closely resembles a real cell membrane in terms of curvature and size [43]. The Langmuir monolayer model has significant advantages in precisely regulating components, surface pressure, environment, and temperature of the membrane system, making it well-suited for investigating the interaction between the lipids and some functional molecules, such as drugs or proteins [44,45,46,47].

In the membrane of human erythrocytes, phospholipids primarily comprise phosphatidylcholine (PC), sphingomyelin, phosphatidylethanolamine, and phosphatidylserine [48]. Among these, PC exhibits the highest abundance [49,50], predominantly located within the external layer of the membrane of erythrocytes [51,52], and it also is a key pulmonary surfactant in the human body [53]. The 1,2-dipalmitoyl-sn-glycero-3-phosphocholine (DPPC) and 1,2-dioleoyl-sn-glycero-3- phosphocholine (DOPC) are respectively saturated and unsaturated phosphatidylcholine, which are commonly employed to simulate the outer leaflet of the cellular membrane [54,55,56]; they are also used to investigate AmB’s adsorption or penetration across the cellular membrane [57,58]. The 1-palmitoyl-2-oleoyl-sn-glycero-3-phosphocholine (POPC) molecules are also commonly used in model membranes, consisting of one unsaturated hydrocarbon chain and one saturated hydrocarbon chain. Compared to POPC molecules, the two tails of a DOPC molecule are unsaturated [59]. Because of the rigid sterol backbone, cholesterol is preferentially positioned in close proximity to saturated hydrocarbon chains of the neighboring lipids, as these are more inflexible and elongated compared with those of unsaturated lipids [34]. In order to allow for the avoidance of potential influences from saturated tails in the results, the DOPC molecules were selected in this work. Therefore, utilizing the DOPC/cholesterol model membrane to investigate the adsorption of AmB on the liquid membrane region is greatly valuable for gaining insights into the mechanism of AmB’s toxicity towards the erythrocyte’s membrane.

According to the literature [60], in the case of 30 patients receiving an oral dose of 1–8 mg/kg per day, the pharmacokinetics of AmB showed that the blood concentration of the drug was approximately 4.9–49.1 μg/mL. To enhance the biological and clinical relevance of the research findings in this work, four different concentrations of AmB were designed to be 5, 45, 85, and 125 μg/mL, respectively, which helped to explore the molecular mechanisms of AmB-induced toxicity to erythrocyte membranes under normal dosage and overdose conditions. The Langmuir monolayer model was utilized for examining the effect of varying the concentration of AmB on the thermodynamic properties and morphology of the DOPC monolayer and the DOPC/cholesterol (1:1) mixed monolayer. The lipid bilayer model was also employed to examine the effects of different concentrations of AmB on the hydrophilic and hydrophobic domains within both the DOPC bilayer and DOPC/cholesterol (1:1) mixed bilayer. The results provide a new insight into the toxic mechanism of the AmB drug on the membrane of erythrocytes at different dosages.

## 2. Results and Discussions

### 2.1. The π−A Isotherm

Molecules arranged in monolayers can exhibit various states based on their density, akin to the behavior seen in three-dimensional systems. They are typically classified as gas, liquid-expanded (LE), liquid-condensed (LC), solid, or transition films [61,62]. The molecular density increases with the increase in surface pressure, which causes a consistent upward trend in the π−A isotherms during compression. The collapse pressure ($\pi_{C}$), liftoff area ($A_{L}$), and limiting area ($A_{\infty }$) are the three key parameters associated with the π−A isotherm. The $\pi_{C}$ refers to the surface pressure at the inflection point at higher surface pressures, meaning the collapse of the monolayer. The $A_{C}$ represents the mean molecular area at this collapse point. The $A_{L}$ refers to the mean molecular area where an initial increase in the π−A isotherm occurs compared to its baseline [50]. Furthermore, the $A_{\infty }$ serves as an empirical parameter that estimates the mean cross-sectional area of molecules [62], which can be calculated from the π−A isotherm by extrapolating the slope of isotherm in its steepest range to the zero-surface pressure [63], especially when a liquid-condensed or solid phase emerges within the isotherm.

**Figure 1:**
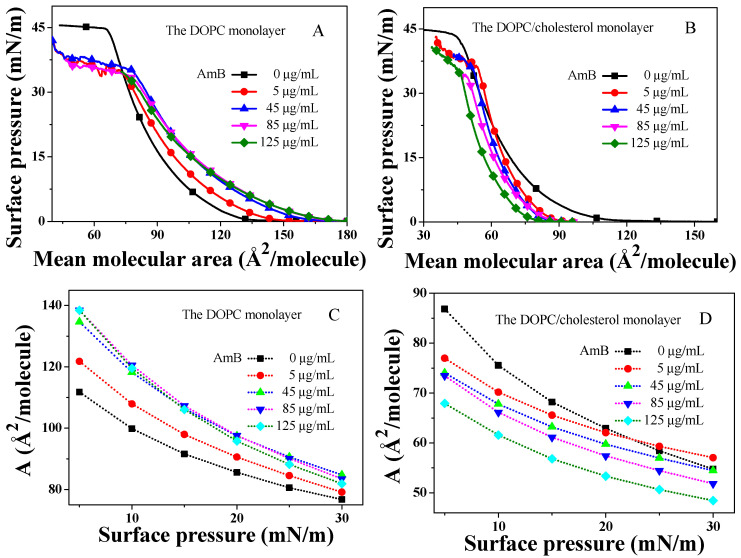
The π−A isotherms of the DOPC monolayer or DOPC/cholesterol (1:1) monolayer with different concentration of AmB.

In the absence of AmB in the buffer, the π−A isotherm of the DOPC monolayer shows a gradual increase starting from approximately 129.8${Å}^{2}$, without any clear phase transition (see Figure 1A), which is consistent with previously documented values in the literature [64]. In contrast, when AmB is present, significant changes are observed in the characteristic parameters of the π−A isotherm for the DOPC monolayer (refer to Table 1). As the concentration of AmB rises, there is a steady increase in the $A_{L}$ value of the DOPC monolayer. Once the concentration of AmB exceeds 85 μg/mL, the $A_{L}$ value stabilizes at around 168.1 ${Å}^{2}$. At the same surface pressure, AmB considerably increases the mean molecular area of the DOPC monolayer (Figure 1C). This indicates that the AmB molecules may be adsorbed from the buffer and inserted into the DOPC monolayer at the interface, which increases the area of the monolayer at the air–water interface. Notably, when the concentration of AmB goes beyond 45 μg/mL, the mean molecular area of the DOPC monolayer changes slightly with the increase in AmB concentration. The insertion of AmB into the DOPC monolayer is related to its concentration. When the concentration of AmB is larger, the amount of AmB involved in inserting into the DOPC monolayer may be close to saturation. The $A_{L}$ value of the DOPC monolayer increases gradually with the increase in AmB concentration (Table 1). The AmB drug significantly decreases the $\pi_{C}$ and $A_{C}$ values of the DOPC monolayer, suggesting that AmB enhances the membrane’s susceptibility to collapse and damage.

In the absence of AmB, the π−A isotherm of the DOPC/cholesterol mixed monolayer exhibits a gradual increase from a mean molecular area of approximately 113.8 ${Å}^{2}$, consistent with that in the literature [65]. In the presence of AmB, the $A_{L}$, $A_{\infty }$, and $A_{C}$ values are all reduced (Figure 1B and Table 1). When the concentration of AmB is greater than 5 μg/mL, the presence of AmB causes the mean molecular area of the DOPC/cholesterol monolayer at the same surface pressure to decrease, indicating that the AmB molecules have not inserted into the DOPC/cholesterol monolayer. Instead, AmB may bind to cholesterol molecules in the interface and remove them from the DOPC/cholesterol monolayer, thus disrupting the integrity of the monolayer. The greater the concentration of AmB, the more significant the decrease in the mean molecular area is. When the AmB concentration is 5 μg/mL, the situation is different from the above. It is worth noting from Figure 1B,D that when the concentration of AmB is 5 μg/mL, the average molecular area of the DOPC/cholesterol monolayer decreases at lower surface pressure. When the DOPC/cholesterol monolayer is compressed to a high surface pressure (21–36 mN/m), the mean molecular area of the DOPC/cholesterol monolayer is larger at the same surface pressure than that in the absence of AmB. This suggests that when the concentration of AmB is 5 μg/mL, at higher surface pressures, AmB molecules are more likely to adsorb and insert into the DOPC/cholesterol monolayer, forming pores (“pore” assumption). At a lower surface pressure, the AmB molecules tend to bind to cholesterol molecules and remove them from the interface, and the AmB–cholesterol complex adheres to the hydrophilic regions of the DOPC/cholesterol monolayer (“sponge” assumption [9]). When the concentration of AmB increases, the mean molecular area of the DOPC/cholesterol monolayer at the same surface pressure gradually decreases, and the $A_{\infty }$ value of the DOPC/cholesterol monolayer also drops significantly. This indicates that as the concentration of AmB increases, the AmB molecules become more likely to remove cholesterol molecules from the DOPC/cholesterol monolayer. The two “pore” and “sponge” mechanisms of AmB are related to the concentration of AmB and the surface pressure of the phospholipid membrane. At high concentrations of AmB, AmB primarily destroys the unsaturated phospholipid/cholesterol monolayer through a “sponge” mechanism, which means that by inhibiting AmB’s ability to extract cholesterol from the phospholipid membrane, the toxicity of this drug to the erythrocyte membrane may be effectively reduced.

### 2.2. Modulus of Elasticity

The modulus of elasticity is an essential factor in defining the compressibility of the monolayer. This value can be calculated using data from a π−A isotherm, as explained in the following function [66,67,68]:

(1) $$C_{s}^{-1}=-A{\left(\partial \pi /\partial A\right)}_{T}$$

where s represents the cross-sectional area of the monolayer, A denotes the mean molecular area, and π refers to the surface pressure. A high modulus of elasticity suggests a less compressible monolayer.

**Figure 2:**
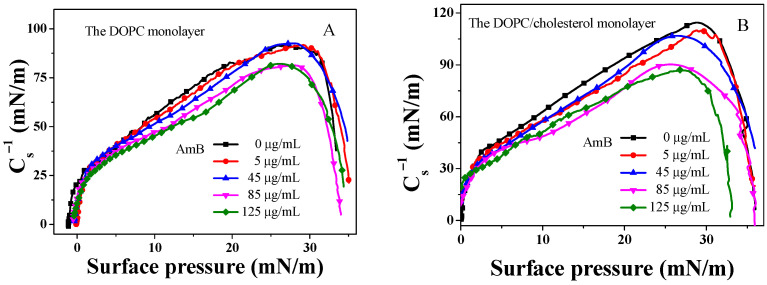
$C_{s}^{-1}$–π curves of DOPC monolayer or DOPC/cholesterol monolayer in the presence of different concentrations of AmB in the buffer.

From Figure 2, the maximum of the $C_{s}^{-1}$ for the DOPC monolayer is 91.7 mN/m, which closely aligns with the values reported in the literature [69]. When the concentration of AmB is 5 μg/mL and 45 μg/mL, the maximum of the $C_{s}^{-1}$ value is almost unchanged. However, when the concentration of AmB is greater than 45 μg/mL, the maximum of the $C_{s}^{-1}$ value decreases significantly. It is worth noting that when the concentration of AmB is 85 μg/mL and 125 μg/mL, the maximum of the $C_{s}^{-1}$ value for the DOPC monolayer is very close. It is evident that when the concentration of AmB is less than 85 μg/mL, the impact of AmB on the maximum modulus of elasticity is weak. However, when the AmB’s concentration exceeds 85 μg/mL, AmB significantly affects the maximum of elastic modulus.

However, in the DOPC/cholesterol monolayer, the maximum of the $C_{s}^{-1}$ value exhibits a decrease with the increase in AmB’s concentration. A reduction of the $C_{s}^{-1}$ value indicates an increased compressibility of the monolayer. The AmB molecules bind to cholesterol in the DOPC/cholesterol monolayer, thereby sequestering cholesterol from the monolayer and increasing the intermolecular distance between the lipid molecules, consequently enhancing the compressibility of the monolayer. This may account for the detrimental effect of AmB on the elasticity of the erythrocyte’s membrane [11].

### 2.3. Adsorption of AmB Molecules on the DOPC Monolayer and the DOPC/Chol Monolayer

**Figure 3:**
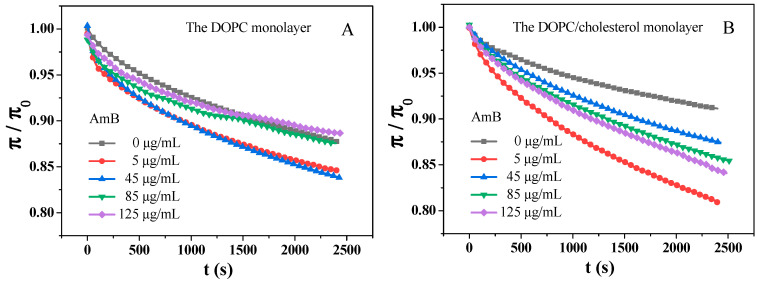
The $\pi /\pi_{0}-t$ curves of the DOPC monolayer (**A**) and DOPC/Chol mixed monolayer (**B**) in the presence of different concentrations of AmB at 37 °C.

At 30 mN/m, the characteristics of lipid monolayers, including surface pressure, molecular area, phase transition, and compressibility, are similar to those in natural bilayers [70]. First, different concentrations of amphotericin B were dissolved in a buffer solution under the air–water interface. The lipid molecules were then spread on the interface to create a lipid monolayer. The monolayer was compressed until it reached a surface pressure of 30 mN/m. The surface pressure–time (π−t) curve at a constant area indicates the relaxation behavior of these monolayers [64]. These π−t curves can be normalized to $\pi /\pi_{0}-t$ curves (Figure 3), which can be accurately fitted using the following equation [61,71]:

(2) $$\pi /\pi_{0}=C+ae^{-t/\tau }$$

where C could be interpreted as the normalized equilibrium pressure and τ could be considered as the lifetime linked to the restructuring of monolayer. As the τ value increases, the relaxation time of the monolayer becomes longer, indicating that the monolayer requires a longer time for conformation transition [61]. The parameter C and τ values of the DOPC monolayer and the DOPC/cholesterol mixed monolayer can be seen in Table 2.

From Table 2, $r^{2}$ represents the degree of fit of the theoretical model formula to the data. The range of $r^{2}$ is between 0 and 1. The closer $r^{2}$ is to 1, the better the data fit. AmB decreases the τ value of the DOPC monolayer, and the greater the concentration of AmB, the stronger the effect. The result shows that the time required for the structure reorganization of the DOPC monolayer at 30 mN/m is shortened due to the presence of AmB. That is, the time required for the surface pressure of the DOPC monolayer to stabilize is shortened. This may be due to the insertion of AmB into the DOPC monolayer, which makes the molecular arrangement at the interface more compact. However, for the DOPC/cholesterol mixed monolayer, when the concentration of AmB is higher than 5 μg/mL, AmB increases the τ value of the monolayer. And the higher the concentration of AmB, the larger the τ value. The higher concentration of AmB results in a longer reorganization time of the DOPC/cholesterol monolayer. This may be due to the cholesterol molecules on the interface being pulled out of the interface by AmB (“sponge” assumption). This causes the tightly packed monolayer to break down, resulting in a longer time for the surface pressure to stabilize. And the more AmB is present, the more significant this effect is. When the concentration of AmB is 5 μg/mL, the τ value of the DOPC/cholesterol monolayer is lower than that without AmB, indicating that low concentration AmB reduces the reorganization time of the DOPC/cholesterol monolayer, which is different from the case when AmB is at other concentrations. According to Figure 1D, at 30 mN/m, when the AmB concentration is 5 μg/mL, the mean molecular area of the DOPC/cholesterol monolayer is greater than that without AmB. The analysis of elastic modulus and the mean molecular area both indicate that the low concentration of AmB interacts with the DOPC/cholesterol monolayer mainly by inserting into the membrane and forming pores (“pore” assumption) at 30 mN/m.

### 2.4. Real Time Morphology of Lipid Monolayer

**Figure 4:**
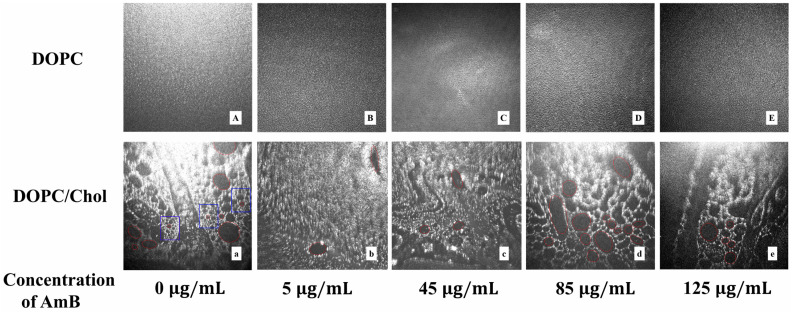
The BAM images (1000 μm × 1000 μm) of the DOPC monolayer (**A**–**E**) or DOPC/cholesterol monolayer (**a**–**e**) in the presence of different concentrations of AmB in the buffer.

The lipid monolayer morphology at the air–water interface can be observed by Brewster angle microscope (BAM) in real time, which reflects the most realistic situation of the monolayer at the air–water interface [72]. After dissolving different concentrations of AmB in the aqueous solution, a lipid monolayer was prepared on the interface and compressed to a surface pressure of 30 mN/m. The surface pressure was kept constant, and after waiting for 5 min, the BAM image of the monolayer was obtained. The bright region is the liquid-condensed phase, and the dark region is the liquid-expanded phase in the BAM images. From Figure 4A–E, the DOPC monolayer is in a homogeneous liquid-condensed phase in the absence of AmB at 30 mN/m. As the concentration of AmB in the buffer increases, the morphology of the DOPC monolayer appears to have changed little, as the image resolution is 12 μm. On the pure water interface, it is observed that the liquid-condensed domain is dispersed homogeneously in the expanded phase in the DOPC/cholesetrol monolayer, which is consistent with the results of previous studies [73,74]. The liquid-condensed phase domain in the DOPC/cholesterol monolayer looks like a network structure and the liquid-expanded phase domain presents an elliptic boundary (the oval red circle is marked in Figure 4a). The domain with the network structure in the blue box of Figure 4 is tight. When the 5 μg/mL AmB is present in the subphase, this network structure is hardly observed, and only sporadic liquid-expanded phases with elliptic borders are observed. It is suggested that AmB molecules may be more inclined to be inserted into the DOPC/cholesterol monolayer, resulting in a more dense molecular arrangement. In the presence of 45 μg/mL AmB, some liquid-expanded phases with elliptic borders are observed, but the network structure of the liquid-condensed domains is not significant. When the concentration of AmB increases to 85 μg/mL, a significant network structure is observed, and the region of the liquid-expanded phases with elliptic borders expands, making this network structure appear looser than that without AmB. The AmB molecules may remove cholesterol molecules from the DOPC/cholesterol monolayer like a “sponge”, which turns a part of the original liquid-condensed phase into the liquid-expanded phase. When the concentration of AmB is 125 μg/mL, part of the network structure becomes a liquid-expanded phase. The higher the concentration of AmB, the stronger the ability to remove cholesterol molecules from the DOPC/cholesterol monolayer. The BAM images confirm that low concentrations of AmB molecules tend to insert into the monolayer at 30 mN/m, making the molecular arrangement more compact. In contrast, high concentrations of AmB molecules tend to remove cholesterol molecules from the interface of the monolayer, causing the liquid-condensed domains formed by cholesterol and DOPC to shrink due to the absence of cholesterol.

### 2.5. Fluorescence Lifetime of NBD Probes in DOPC Liposomes and DOPC/Cholesterol Liposomes

The fluorescence lifetime can accurately and effectively reflect the microenvironment of the fluorescent probe at its location [75]. The fluorescence lifetime of the NBD group is very sensitive to the microenvironment around it [76]. Therefore, studying the fluorescence lifetime of NBD groups at different locations can help investigate the effects of different concentrations of AmB on different regions in the bilayer.

According to Formula 3, the fluorescence intensity decay curves (Figures S1 and S2) were fitted to obtain the fluorescence lifetime of the NBD-PE and 6-NBD-PC in the bilayer of DOPC liposomes and DOPC/Chol liposomes in the presence of different concentrations of AmB, as shown in Table 3. The average fluorescence lifetime was calculated using Formula 4.

**Figure 5:**
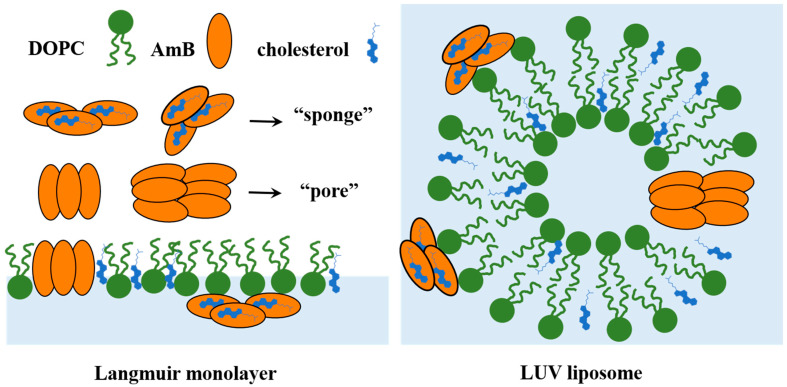
A diagram of the isolation of cholesterol caused by AmB from the lipid membrane.

From Table 3, it can be seen that the average lifetime of the fluorescent probe has shortened, indicating an increase in the disorder of the bilayer. The increase in the average lifetime indicates a decrease in disorder in the bilayer. For pure DOPC liposomes, the average fluorescence lifetime of the NBD-PE probe and the NBD-PC probe all gradually increase with the rise in AmB’s concentration. It is indicated that the disorder of the hydrophilic and hydrophobic regions of the bilayer decreases with the increase in AmB’s concentration. It may be inferred that the AmB molecules mainly insert into the DOPC bilayer so that the molecules on the interface are arranged more closely. For the DOPC/cholesterol liposomes, when the concentration of AmB is 5 μg/mL, the average fluorescence lifetime of the NBD-PC probe is larger than that in the absence of AmB. This means that the presence of 5 μg/mL AmB leads to a more ordered hydrophobic region in the bilayer, possibly due to the insertion of AmB molecules. When the concentration of AmB is greater than 5 μg/mL, the average lifetime of the NBD-PC probe decreases with the rise in AmB, indicating that the hydrophobic domain of the DOPC/cholesterol bilayer becomes more disordered as the concentration of AmB increases. This agrees with the relaxation time analysis of the monolayer. However, for the DOPC/cholesterol liposomes, the average lifetime of the NBD-PE probe continuously decreases with the increase in AmB’s concentration, and there was no particularity at AmB concentration of 5 μg/mL. This suggests that AmB increases the disorder of the hydrophilic region in the DOPC/cholesterol bilayer, and the higher concentration of AmB corresponds to a stronger degree of the influence. This may be due to the cholesterol molecules being pulled out of the bilayer (“sponge” assumption) by AmB (Figure 5), which causes the molecular arrangement on the bilayer to become disordered, which is also consistent with the relaxation time analysis of the monolayer model. The more AmB concentration, the more AmB molecules involved in isolating cholesterol molecules, which causes the disorder of the hydrophilic domain of the DOPC/cholesterol bilayer to be greater.

## 3. Materials and Methods

### 3.1. Materials

**Figure 6:**
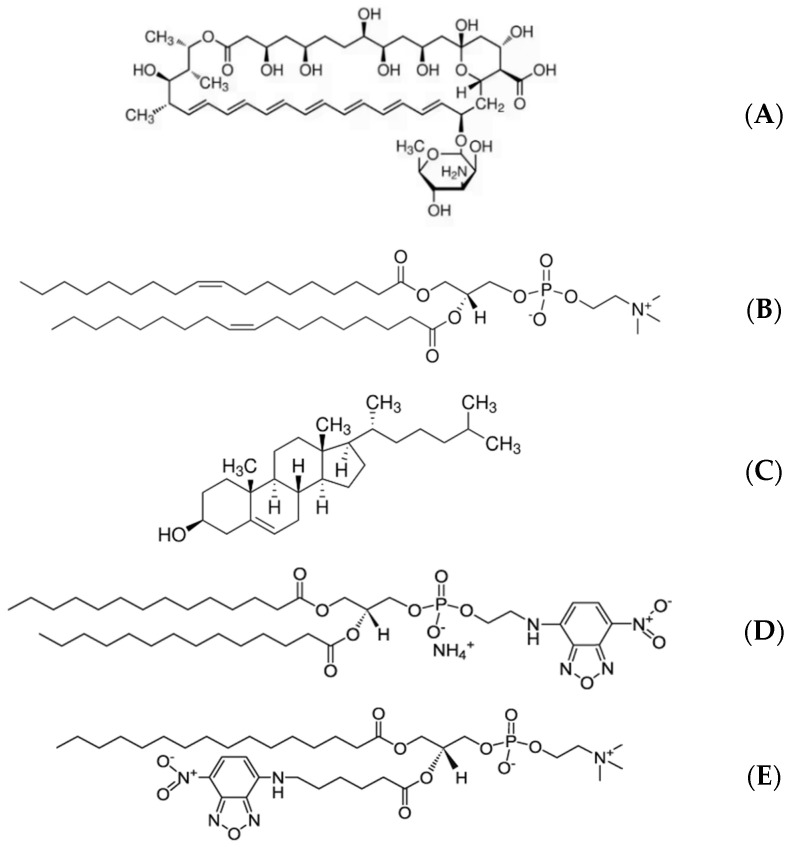
The molecular structures of AmB (**A**), DOPC (**B**), cholesterol (**C**), NBD-PE (**D**), and 6-NBD-PC (**E**).

1,2-Dioleoyl-sn-glycero-3-phosphocholine (DOPC; 99% purity), cholesterol (Chol; 99% purity), and soluble AmB (19.8 mg/mL) were obtained from Sigma, St. Louis, MO, USA, with their structures depicted in Figure 6. The pure DOPC molecules and the DOPC/cholesterol mixture at a molar ratio of 1:1 were solubilized in a solvent combination of chloroform and methanol with a volume ratio of 9:1, achieving final concentrations of 0.5 mM for both the pure DOPC solution and the mixed DOPC/cholesterol solution. The aqueous phase in the trough was created using a stable pH 7.0 buffer made up of 20 mM HEPES (N-2-hydroxyethylpiperazine-N-2-ethanesulfonic acid). The AmB solutions were diluted with HEPES buffer to prepare the subphase solution containing AmB concentrations of 5, 45, 85, and 125 µg/mL for experimental use. In every experiment, high-purity water was employed, and it is sourced from a Milli-Q Plus purification system (18.2 MΩ/cm; Millipore, Bedford, MA, USA).

### 3.2. Langmuir Monolayer Experiment

The Langmuir trough (KSV-Minitrough, Helsinki, Finland) provides an air–water interface. Two Teflon barriers are used to control the compression or expansion of the monolayer at a specified rate. A Wilhelmy-type surface tension meter measures changes in the surface pressure of the monolayer with a precision of 0.1 mN/m during this process. To begin, both the Teflon trough and barriers were cleaned with ethanol to remove any possible contaminants. Next, 200 mL of HEPES solution or HEPES solution containing different concentrations of AmB was added to the trough. After that, a Hamilton micropipette dispensed 20 µL of DOPC solution or the mixture of DOPC/cholesterol onto the air–water interface; then, after 10 min, the organic solvent evaporated. The monolayer was compressed at a rate of 7 mm/min while monitoring the surface pressure versus mean molecular area (π−A) isotherm. When the surface pressure of the monolayer reached 30 mN/m, compression was stopped and the area was kept constant. Then, the curve of the surface pressure over time was recorded. All experiments were performed three times to ensure reproducibility, with an external circulator keeping the temperature stable at 37 ± 0.5 °C.

### 3.3. The Real Time Morphology of Monolayer by BAM

The Brewster angle microscope (BAM, KSV NIMA, Helsinki, Finland) can be used to observe the morphology lipid monolayer at the air–water interface in real time without the need for fluorescent labels. This microscope utilizes a 50 mW laser that produces p-polarized light with a wavelength of 659 nm. Initially, the reflector was placed beneath the air–water interface within the subphase solution. The lipid molecules were spread over this interface, and the monolayer was compressed to make the surface pressure reach the target value. Finally, the morphology was observed using the Brewster angle microscope positioned above the reflector. The image resolution was 12 μm, and images were captured with a field size of 1000 μm × 1000 μm in this work.

### 3.4. Preparation of Large Unilamellar Vesicle (LUV) Liposomes

Large unilamellar vesicle (LUV) liposomes were prepared by the ultrasonic thin film dispersion method. Fluorescence experiments were performed with a 200 nm diameter 2 mol% NBD labeled lipid probe using either DOPC liposomes or DOPC/cholesterol liposomes. The NBD probes used NBD-PE and 6-NBD-PC [77]. The NBD group is located on the polar head of the NBD-PE molecule, while the NBD group in 6-NBD-PC is located on the acyl chain. The lifetime of the two fluorescence probes can be used to probe the disorder in the hydrophilic and hydrophobic regions of the membrane.

The preparation method is as follows. The lipid molecules and the NBD probes were dissolved in chloroform, mixed thoroughly, poured into a round-bottom flask, and placed on a vacuum rotary evaporator at 37 °C to dry. For the mixed lipids, the molar ratio of DOPC and cholesterol was 1:1. Then, the round-bottom flask was placed in a vacuum drying oven and dried thoroughly at 37 °C for 12 h. Next, 10 mL of 20 mM HEPES buffer was added to the round-bottom flask using a rotary evaporator to hydrate the lipid molecules under normal pressure, resulting in a liposome solution. Then, the liposome solution was sonicated in an ice bath using a sonic cell disrupter for 5 min (working 1 s, pausing 2 s, power 200 W) to reduce the particle size, and filtered through a 200 nm polycarbonate filter membrane 3 times to obtain dispersed large unilamellar liposomes. A control sample without the fluorescent probe was also prepared using the same method. Before the fluorescence test, different amounts of AmB were added to the sample solution to achieve AmB concentrations of 5, 45, 85, and 125 µg/mL in the liposome solution, and allowed to stand for 1 h.

### 3.5. Time-Resolved Fluorescence Measurements

Fluorescence measurements with time resolution were performed using the Fluorescence Master Systems (PTI, Edison, NJ, USA). The excitation wavelength was established at 470 nm, and the emission wavelength was measured at 540 nm. All lifetimes were calibrated using POPOP as a reference standard (1.35 ns). Each experiment employed excitation and emission slits with a bandpass of 5 nm. The decay curve of fluorescence intensity was fitted to the following exponential equation [78,79]:

(3) $$F(t)=\sum \alpha_{i}exp(-t/\tau_{i})$$

where F(t) denotes the fluorescence intensity at time t, and $\alpha_{i}$ represents the pre-exponential factor that quantifies the contribution of each lifetime component. The average lifetime of the bi-exponential decay fluorescence was calculated by the decay time and the pre-exponential factor, as shown by the formula below [78,79]:

(4) $$<\tau >=\frac{\alpha_{1}\tau_{1}^{2}+\alpha_{2}\tau_{2}^{2}}{\alpha_{1}\tau_{1}+\alpha_{2}\tau_{2}}$$

## 4. Conclusions

This work studied the effects of different concentrations of AmB on the π−A isotherm, elastic modulus, relaxation time, and morphology of the unsaturated phospholipid membrane and the unsaturated phospholipid/cholesterol (1:1) mixed membrane using the Langmuir monolayer model. This study also investigated the effects of different concentrations of AmB on the orderedness of the hydrophilic and hydrophobic domains of these lipid bilayers by two NBD fluorescence probes. AmB increases the mean molecular area of the DOPC monolayer, and the greater the concentration of AmB, the greater this effect. The molecules on the DOPC membrane are more densely packed due to the presence of AmB, as evidenced by the analysis of the relaxation time of the DOPC monolayer and the fluorescence lifetime of the fluorescence probe in the DOPC bilayer. This may be due to the insertion of AmB molecules into the membrane, which causes the molecules in the membrane to arrange more closely. However, the mean molecular area of the DOPC/cholesterol monolayer affected by AmB depends on the concentration of AmB. When the concentration of AmB exceeds 5 μg/mL, the greater the drug’s concentration, the more significant the reduction in the mean molecular area of the DOPC/cholesterol monolayer, and the stronger the disorder of the hydrophobic region in the DOPC/cholesterol mixed membrane, which has been confirmed in studies of both the monolayer model and the bilayer model. This may be due to the fact that the cholesterol molecules are pulled out of the bilayer (the “sponge” hypothesis) by AmB, leading to a more disordered arrangement of molecules on the membrane. When the concentration of AmB is 5 μg/mL, the mean molecular area of the DOPC/cholesterol monolayer decreases at a lower surface pressure. But at a higher surface pressure, the mean molecular area of the DOPC/cholesterol monolayer increases, and the disorder of the hydrophobic regions in the DOPC/cholesterol mixed membrane decreases. At a low concentration (5 and 45 μg/mL) of AmB, this drug has a weaker effect on the maximum value of elastic modulus for the DOPC/cholesterol monolayer. The way in which AmB interacts with the DOPC/cholesterol mixed membrane is related to the concentration of AmB. The higher the concentration of AmB, the more likely it is to remove cholesterol from the unsaturated phospholipid membrane, which is similar to the result in the literature [80] and is evidenced by the BAM images.

The results have a reference value for understanding the toxic mechanism of AmB on the liquid membrane region composed of unsaturated phospholipid and cholesterol in the erythrocyte’s membrane, at a normal and excessive dosage of AmB. The information in the results is helpful to understand the mechanism of AmB’s toxicity to erythrocyte membranes at the membrane level, and has guiding value for seeking ways to reduce the toxicity. However, the actual components of the cell membrane are complex, so it is necessary to use multiple different phospholipid membrane systems to find out the mechanism of action of AmB. In the future, it can also be obtained by neutron scattering that the structure of the membrane system is affected by AmB.

## Figures and Tables

**Table 1:** The $A_{L}$, $A_{\infty }$, $\pi_{C}$, and $A_{C}$ values of the DOPC monolayer or DOPC/cholesterol monolayer with different concentration of AmB.

Monolayer	Different Concentration of AmB (μg/mL)	$A_{L}/{Å}^{2}$	$A_{\infty }/{Å}^{2}$	$\pi_{C}/mN/m$	$A_{C}/{Å}^{2}$
DOPC	0	129.8 ± 0.5	98.5 ± 0.3	44.5 ± 0.1	65.9 ± 0.1
	5	143.5 ± 0.4	110.5 ± 0.2	34.2 ± 0.1	50.0 ± 0.2
	45	158.2 ± 0.6	120.4 ± 0.2	34.7 ± 0.2	58.4 ± 0.3
	85	168.1 ± 0.2	121.2 ± 0.5	33.1 ± 0.1	56.7 ± 0.4
	125	168.1 ± 0.4	121.7 ± 0.4	34.2 ± 0.2	54.7 ± 0.1
DOPC/Chol (1:1)	0	113.8 ± 0.1	74.3 ± 0.2	43.1 ± 0.1	44.6 ± 0.4
	5	88.3 ± 0.1	72.2 ± 0.1	35.7 ± 0.3	54.6 ± 0.2
	45	88.0 ± 0.1	70.4 ± 0.2	35.4 ± 0.1	52.2 ± 0.4
	85	87.1 ± 0.3	68.7 ± 0.2	33.1 ± 0.2	49.4 ± 0.1
	125	82.2 ± 0.2	64.0 ± 0.1	33.9 ± 0.2	47.1 ± 0.3

**Table 2:** The $C,\;a,\;\tau ,\;r^{2}$ values of the decay curves fitted by a single-exponential equation.

Monolayer	Different Concentrations of AmB (μg/mL)	C	a	τ	$r^{2}$
DOPC	0	0.848 ± 0.011	0.144 ± 0.001	1603.752 ± 5.119	0.997 ± 0.002
	5	0.820 ± 0.008	0.150 ± 0.001	1427.784 ± 8.214	0.992 ± 0.006
	45	0.813 ± 0.012	0.164 ± 0.002	1396.158 ± 5.124	0.993 ± 0.005
	85	0.863 ± 0.011	0.111 ± 0.001	1274.915 ± 6.124	0.988 ± 0.002
	125	0.878 ± 0.011	0.105 ± 0.001	1094.702 ± 5.112	0.985 ± 0.001
DOPC/Chol (1:1)	0	0.888 ± 0.021	0.105 ± 0.003	1685.954 ± 4.125	0.996 ± 0.007
	5	0.761 ± 0.019	0.222 ± 0.002	1660.208 ± 5.142	0.997 ± 0.003
	45	0.835 ± 0.013	0.159 ± 0.002	1796.342 ± 10.241	0.998 ± 0.001
	85	0.807 ± 0.011	0.183 ± 0.001	1933.925 ± 9.145	0.996 ± 0.001
	125	0.780 ± 0.011	0.207 ± 0.003	2145.859 ± 11.245	0.996 ± 0.002

**Table 3:** Fluorescence lifetimes and apparent rotational correlation times of NBD-PE and 6-NBD-PC in the membrane of DOPC liposomes and DOPC/Chol (1:1) liposomes in the solution containing 0, 5, 45, 85, and 125 μg/mL AmB.

Lipid	AmB μg/mL	NBD-PE						NBD-PC					
		α_1_	τ_1_/ns	α_2_	τ_2_/ns	<τ>/ns	χ^2^	α_1_	τ_1_/ns	α_2_	τ_2_/ns	<τ>/ns	χ^2^
DOPC	0	10.79 ± 0.03	14.16 ± 0.02	3.28 ± 0.01	2.91 ± 0.02	13.50 ± 0.01	0.96	1.83 ± 0.03	19.43 ± 0.02	7.56 ± 0.08	2.41 ± 0.02	13.66 ± 0.01	0.96
	5	7.74 ± 0.08	13.55 ± 0.03	7.74 ± 0.03	13.55 ± 0.01	13.55 ± 0.01	0.92	0.19 ± 0.01	39.96 ± 0.05	5.69 ± 0.04	2.69 ± 0.01	15.05 ± 0.02	0.96
	45	2.11 ± 0.01	18.61 ± 0.06	3.00 ± 0.03	2.79 ± 0.11	15.83 ± 0.03	0.96	4.32 ± 0.06	17.90 ± 0.03	4.62 ± 0.01	3.03 ± 0.01	15.62 ± 0.02	0.98
	85	3.57 ± 0.05	20.89 ± 0.11	2.63 ± 0.02	3.47 ± 0.06	18.99 ± 0.01	0.93	0.57 ± 0.01	33.69 ± 0.03	1.50 ± 0.01	2.61 ± 0.01	28.43 ± 0.05	0.97
	125	0.73 ± 0.02	27.75 ± 0.10	1.50 ± 0.01	3.11 ± 0.01	23.14 ± 0.01	0.95	0.15 ± 0.01	127.27 ± 0.09	5.59 ± 0.02	2.23 ± 0.02	77.88 ± 0.11	0.97
DOPC-Chol (1:1)	0	44.27 ± 0.10	12.80 ± 0.08	7.73 ± 0.02	1.58 ± 0.01	12.56 ± 0.01	0.95	0.26 ± 0.01	26.70 ± 0.03	4.71 ± 0.02	2.80 ± 0.01	11.04 ± 0.01	0.83
	5	25.37 ± 0.09	11.21 ± 0.01	25.37 ± 0.01	11.21 ± 0.08	11.21 ± 0.01	0.95	28.11 ± 0.03	11.21 ± 0.05	1.12 ± 0.01	2.57 ± 0.01	11.13 ± 0.01	0.95
	45	282.22 ± 0.11	8.20 ± 0.02	3.00 ± 0.01	2.09 ± 0.01	8.18 ± 0.03	0.96	882.17 ± 0.10	6.79 ± 0.03	1.08 ± 0.01	1.97 ± 0.02	6.79 ± 0.02	0.87
	85	1621.60 ± 0.10	6.89 ± 0.02	1.18 ± 0.01	3.49 ± 0.01	6.89 ± 0.03	0.90	2.73 ± 0.04	3.69 ± 0.01	2.73 ± 0.01	3.69 ± 0.02	3.69 ± 0.01	0.82
	125	0.80 ± 0.01	31.48 ± 0.08	3728.90 ± 0.13	6.68 ± 0.04	6.71 ± 0.05	0.98	6.51 ± 0.03	3.49 ± 0.01	6.51 ± 0.04	3.49 ± 0.03	3.49 ± 0.01	0.89

## Data Availability

Data are contained within the article and Supplementary Materials.

## Supplementary Materials

The following supporting information can be downloaded at: https://www.mdpi.com/article/10.3390/molecules29235659/s1, Figure S1: Fluorescence lifetime curves of 6-NBD-PC probe in DOPC and DOPC/Chol liposomes were fitted at AmB concentrations of 0 μg/mL (A,a), 5 μg/mL (B,b), 45 μg/mL(C,c), 85 μg/mL(D,d), and 125 μg/mL (E,e); Figure S2: Fluorescence lifetime curves of NBD-PE probe in DOPC and DOPC/Chol liposomes were fitted at AmB concentrations of 0 μg/mL (A,a), 5 μg/mL (B,b), 45 μg/mL(C,c), 85 μg/mL (D,d), and 125 μg/mL (E,e).
