# Supplementary material for: Effects of Different Concentrations of AmB on the Unsaturated Phospholipid–Cholesterol Membrane Using the Langmuir Monolayer and Liposome Models
Source: Molecules. 2024 Nov 29;29(23):5659. doi: 10.3390/molecules29235659 (PMC11644019; doi:10.3390/molecules29235659)
Supplement: Supplementary file 1 [file molecules-29-05659-s001.zip › molecules-3236506-supplementary.pdf]

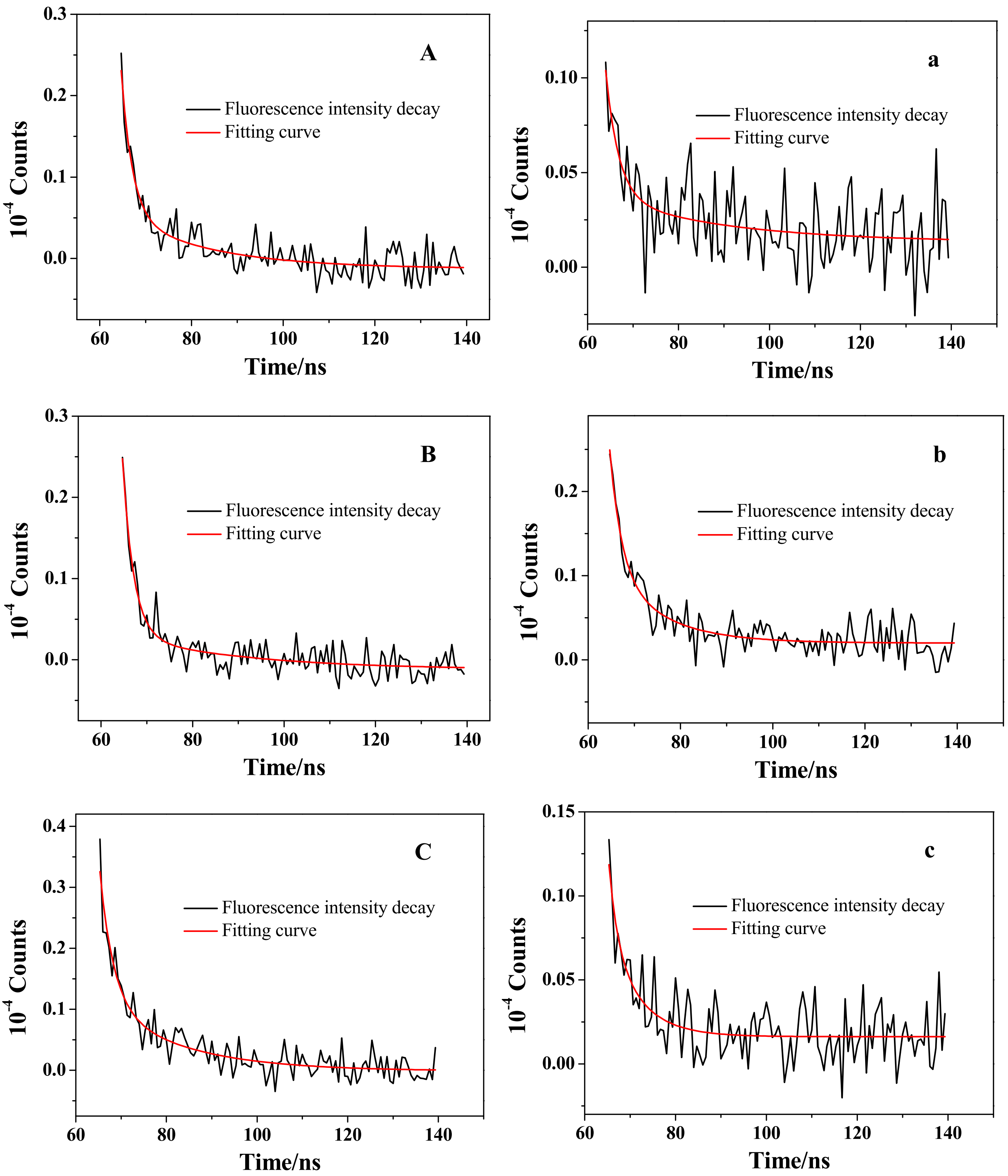

A
a
B
b
C
c
Fluorescence intensity decay
Fitting curve
10^{-4} Counts
Time/ns

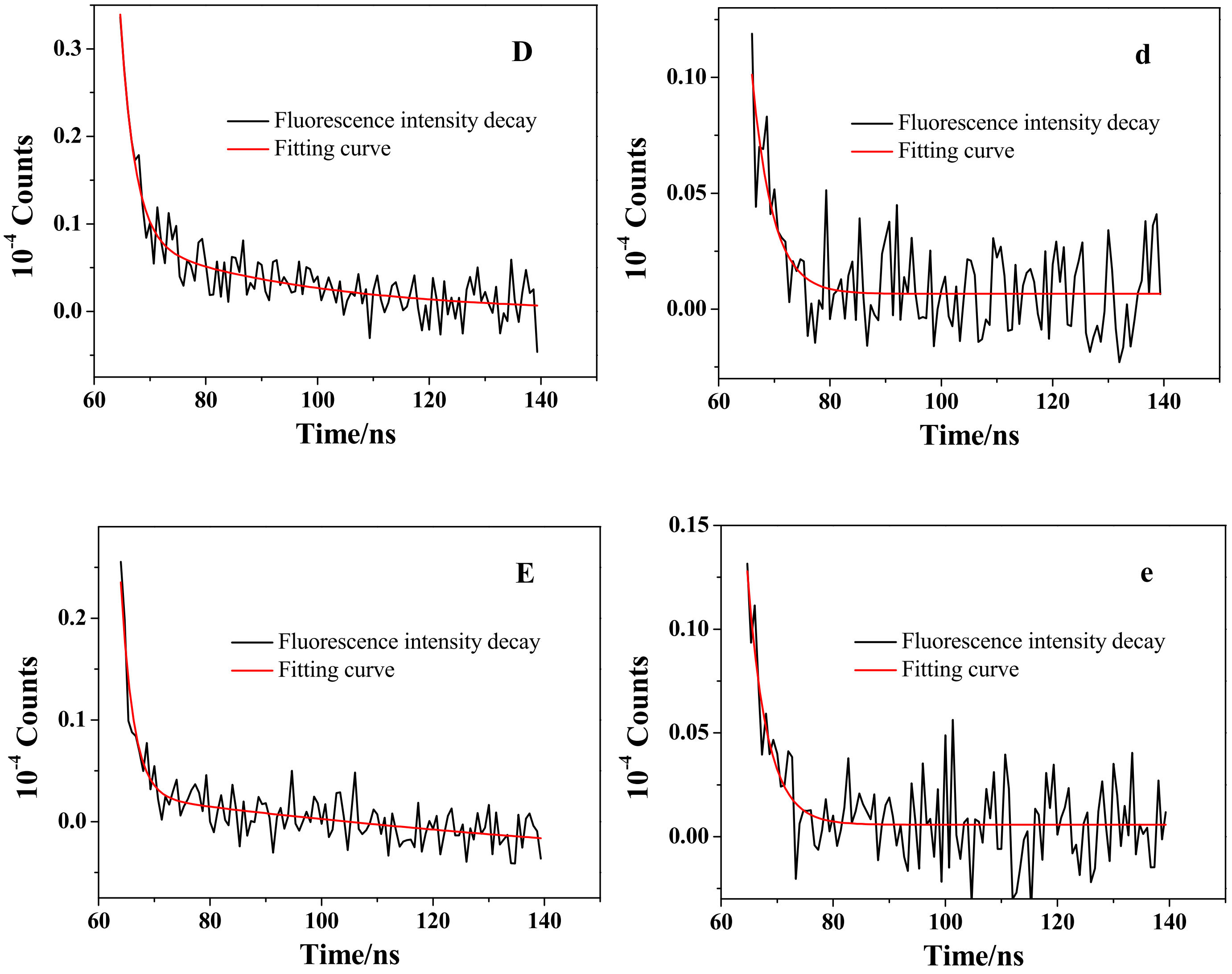

Figure S1 Fluorescence lifetime curves of 6-NBD-PC probe in DOPC and DOPC/Chol liposomes were fitted at AmB concentrations of $0\mu g/mL$（A, a）, $5\mu g/mL$（B, b）, $45\mu g/mL$（C, c）, $85\mu g/mL$（D, d）, $125\mu g/mL$（E, e）.

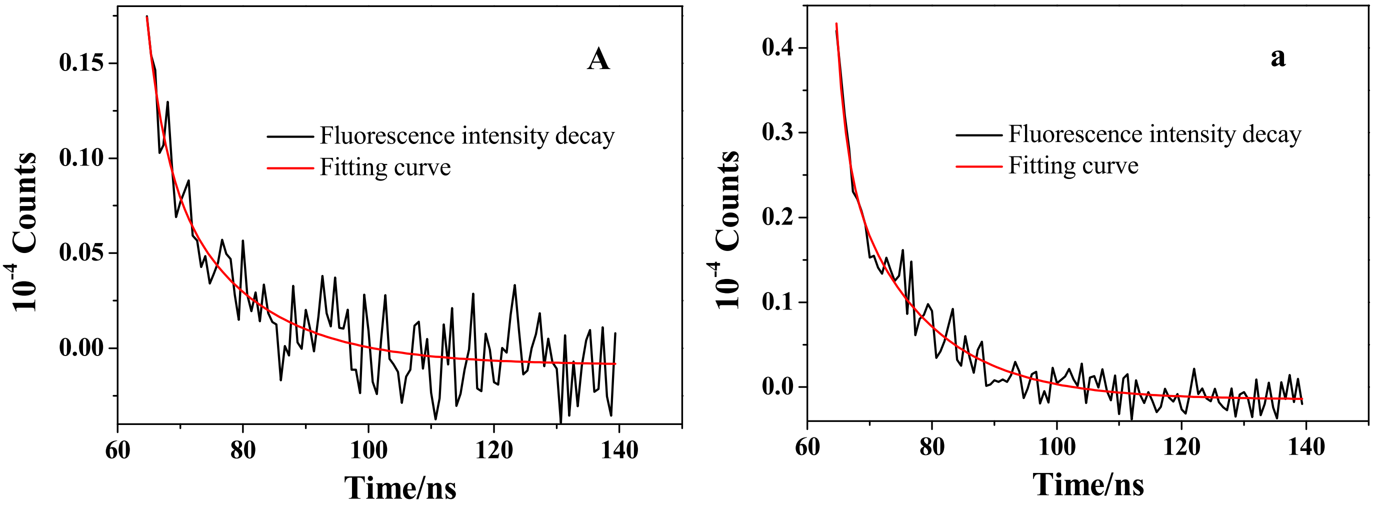

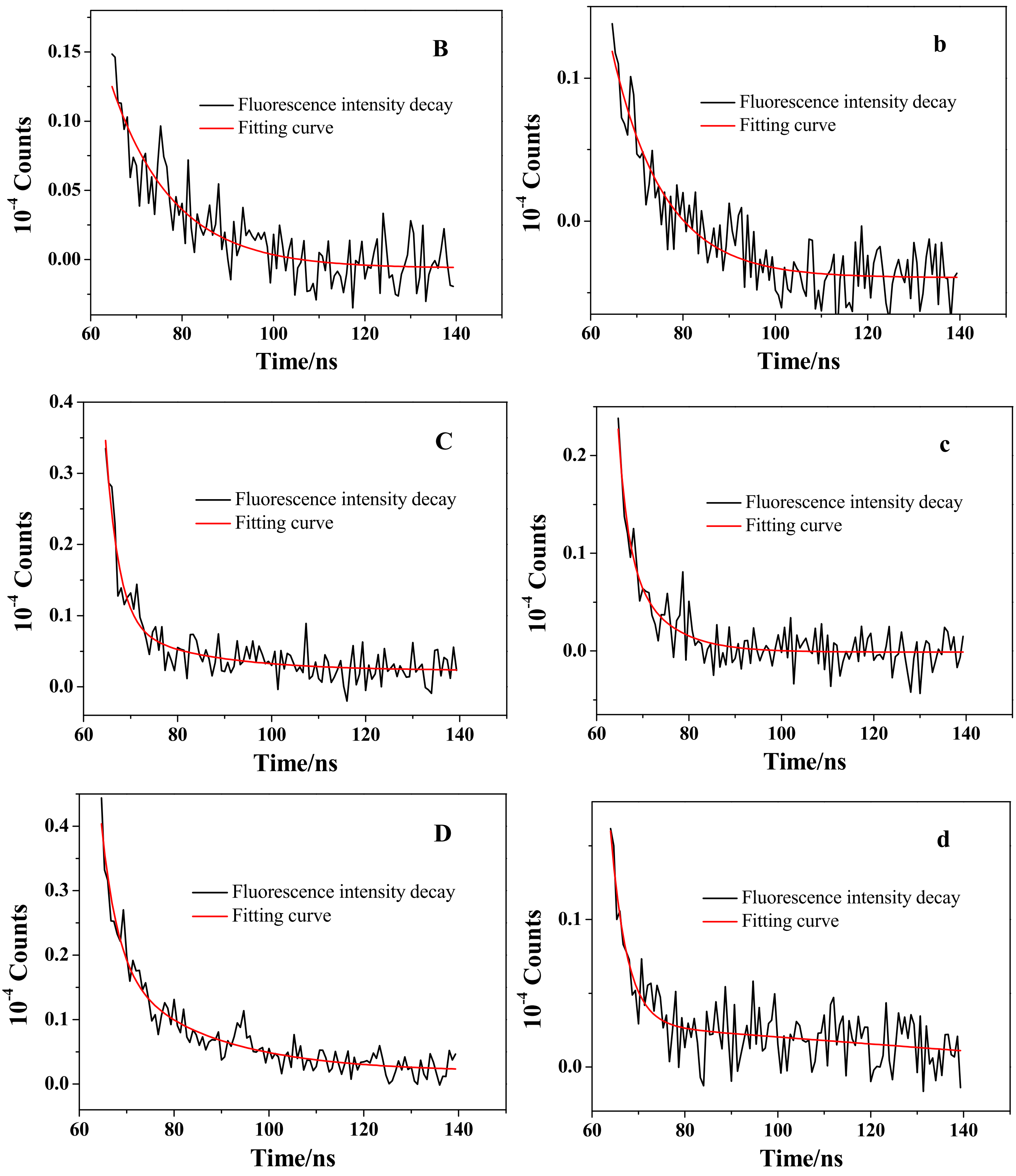

B
Fluorescence intensity decay
Fitting curve
10^{-4} Counts
Time/ns
b
Fluorescence intensity decay
Fitting curve
10^{-4} Counts
Time/ns
C
Fluorescence intensity decay
Fitting curve
10^{-4} Counts
Time/ns
c
Fluorescence intensity decay
Fitting curve
10^{-4} Counts
Time/ns
D
Fluorescence intensity decay
Fitting curve
10^{-4} Counts
Time/ns
d
Fluorescence intensity decay
Fitting curve
10^{-4} Counts
Time/ns

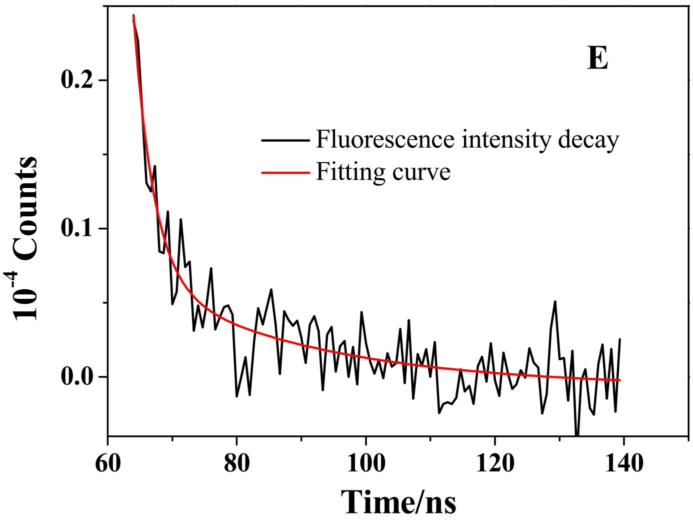

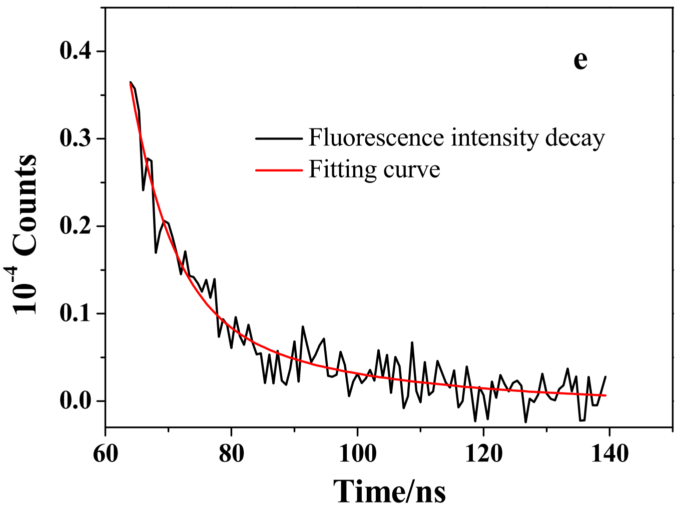

Figure S2 Fluorescence lifetime curves of NBD-PE probe in DOPC and DOPC/Chol liposomes were fitted at AmB concentrations of $0\mu g/mL$（A, a）, $5\mu g/mL$（B, b）, $45\mu g/mL$（C, c）, $85\mu g/mL$（D, d）, $125\mu g/mL$（E, e）.
